# Genome Sequence of *Bacillus* Phage Saddex

**DOI:** 10.1128/MRA.01044-18

**Published:** 2018-09-20

**Authors:** Elizabeth Greguske, Alexis Nadeau, Emily Fitzmeyer, Karolina Fucikova

**Affiliations:** a Department of Biology, Saint Anselm College, Manchester, New Hampshire, USA; b Department of Natural Science, Assumption College, Worchester, Massachusetts, USA; Georgia Institute of Technology

## Abstract

The complete genome of Bacillus phage Saddex was determined and annotated in this study. Saddex has distinct sections with similarities to other Bacillus phages, such as Kida, even though these phages were isolated more than 800 km apart by separate laboratories.

## ANNOUNCEMENT

Saddex is a novel Bacillus bacteriophage isolated, characterized, and annotated by students in the Howard Hughes Medical Institute (HHMI) Phage Hunters program. Saddex is able to lyse multiple Bacillus host species, including Bacillus cereus, which is of particular interest, as this bacterium lives in the gut of poultry as well as in soil and causes an estimated 2% of all cases of food poisoning (1).

Saddex was isolated from lawn soil sampled (at coordinates N42.791838, W71.069913) in Haverhill, Massachusetts. In brief, log phase Bacillus thuringiensis subspecies *kurstaki* cells were mixed with lawn soil and allowed to grow overnight while being shaken at 37°C. Phage were isolated via centrifugation for 10 min at 3,000 rpm, followed by filtering with a 0.22-µm sterile syringe filter (2). DNA was isolated from purified phage with the Qiagen viral DSP spin kit version 1 and sequenced via the HiSeq 2500 Illumina platform at the Hubbard Center for Genome Studies (University of New Hampshire, Durham, NH), resulting in 779,058 paired-end reads and an average 250-bp read length. Reads were trimmed with Trimmomatic (3), assembled into contigs with QUAST (4), and then refined with Geneious version 10.2 (5) reference assemblies with custom low sensitivity (allowing only 2% mismatches for precise mapping). The average depth of coverage was 539.4× with no areas of poor coverage noted. Saddex was autoannotated in Geneious version 10.2 with default settings, with a known Bacillus phage genome, Evoli (GenBank accession number KJ489398), for comparison. The genome was then visually cross-checked against 11 other complete Bacillus phage genomes available in GenBank.

The complete genome of Saddex is 142,353 bp of linear, double-stranded DNA with a G+C content of 39.0%. All genes in Saddex were found to have at least one homolog in other published Bacillus phages (2, 6–10) with a BLAST nucleotide (BLASTn) analysis (11), indicating that no novel genes were identified. However, the nucleotide sequence identity similarity of these genes ranged from as low as 63% to as high as 99%, with an average 81.9% similarity; this shows that Saddex could have unique polymorphisms. Of the 208 predicted proteins, 54 were assigned a function, typically for tail and capsid structure, nuclease activity, and lytic activity. Three tRNAs were identified, all three of which were found to have 100% similarity to at least one other Bacillus phage with a BLASTn analysis (11).

The proposed Bacillus phage cluster guidelines (12), in which pairwise average nucleotide identity across the genome is used to group similar phage, place Saddex in the C1 cluster of Bacillus phages.

### Data availability.

The complete genome sequence of the Bacillus phage Saddex is available in GenBank under accession number MH538193. Raw reads are available in the SRA under accession number SRP158918.

## References

[1] Kotiranta A, Lounatmaa K, Haapasalo M 2000 Epidemiology and pathogenesis of *Bacillus cereus* infections. Microbes Infect 2:189–198. doi:10.1016/S1286-4579(00)00269-0. 10742691

[2] Sauder AB, Quinn MR, Brouillette A, Caruso S, Cresawn S, Erill I, Lewis L, Loesser-Casey K, Pate M, Scott C, Stockwell S, Temple L 2016 Genomic characterization and comparison of seven *Myoviridae* bacteriophage infecting *Bacillus thuringiensis*. Virology 489:243–251. doi:10.1016/j.virol.2015.12.012. 26773385

[3] Bolger AM, Lohse M, Usadel B 2014 Trimmomatic: a flexible trimmer for Illumina sequence data. Bioinformatics 30:2114–2120. doi:10.1093/bioinformatics/btu170. 24695404 PMC4103590

[4] Gurevich A, Saveliev V, Vyahhi N, Tesler G 2013 QUAST: quality assessment tool for genome assemblies. Bioinformatics 29:1072–1075. doi:10.1093/bioinformatics/btt086. 23422339 PMC3624806

[5] Kearse M, Moir R, Wilson A, Stones-Havas S, Cheung M, Sturrock S, Buxton S, Cooper A, Markowitz S, Duran C, Thierer T, Ashton B, Meintjes P, Drummond A 2012 Geneious Basic: an integrated and extendable desktop software platform for the organization and analysis of sequence data. Bioinformatics 28:1647–1649. doi:10.1093/bioinformatics/bts199. 22543367 PMC3371832

[6] Cornell JL, Breslin E, Schuhmacher Z, Himelright M, Berluti C, Boyd C, Carson R, Del Gallo E, Giessler C, Gilliam B, Heatherly C, Nevin J, Nguyen B, Nguyen J, Parada J, Sutterfield B, Tukruni M, Temple L 2016 Complete genome sequence of *Bacillus thuringiensis* bacteriophage Smudge. Genome Announc 4:e00572-16. doi:10.1128/genomeA.00572-16. 27540049 PMC4991694

[7] Lorenz L, Lins B, Barrett J, Montgomery A, Trapani S, Schindler A, Christie GE, Cresawn SG, Temple L 2013 Genomic characterization of six novel *Bacillus pumilus* bacteriophages. Virology 444:374–383. doi:10.1016/j.virol.2013.07.004. 23906709

[8] Reveille AM, Eldridge KA, Temple LM 2016 Complete genome sequence of *Bacillus megaterium* bacteriophage Eldridge. Genome Announc 4:e01728-1. doi:10.1128/genomeA.01728-15. 27103735 PMC4841150

[9] Sauder AB, Carter B, Langouet Astrie C, Temple L 2014 Complete genome sequence of a mosaic bacteriophage, Waukesha92. Genome Announc 2:e00339-14. doi:10.1128/genomeA.00339-14. 25146131 PMC4153491

[10] Flounlacker K, Miller R, Marquez D, Johnson A; 2015–2016 VCU Phage Hunters. 2017 Complete genome sequences of *Bacillus* phages DirtyBetty and Kida. Genome Announc 5:e01385-16. doi:10.1128/genomeA.01385-16. PMC5347238 28280018

[11] Altschul SF, Gish W, Miller W, Myers EW, Lipman DJ 1990 Basic local alignment search tool. J Mol Biol 215:403–410. doi:10.1016/S0022-2836(05)80360-2. 2231712

[12] Grose JH, Jensen GL, Burnett SH, Breakwell DP 2014 Genomic comparison of 93 *Bacillus* phages reveals 12 clusters, 14 singletons and remarkable diversity. BMC Genomics 15:855. doi:10.1186/1471-2164-15-855. 25280881 PMC4197329

